# Supplementary material for: Comparative multiomics analysis of cell physiological state after culture in a basket bioreactor
Source: Sci Rep. 2022 Nov 23;12:20161. doi: 10.1038/s41598-022-24687-4 (PMC9686226; doi:10.1038/s41598-022-24687-4)
Supplement: Supplementary file 1 — Supplementary Information 1. [file 41598_2022_24687_MOESM1_ESM.zip › raw data/Proteomic raw data/6.KEGGenrichment/EG.vs.CG/DOWN/pathway/src/00565.html]

map00565


- K01080, 3.1.3.4, R04162"

- Down regulated proteins

A0A0D9S760(Description:Phospholipid phosphatase 3;Gene:PLPP3;log2FC:-1.3288)

Close
